# Dynamics of Diversity and Abundance of Sulfonamide Resistant Bacteria in a Silt Loam Soil Fertilized by Compost

**DOI:** 10.3390/antibiotics10060699

**Published:** 2021-06-11

**Authors:** Hui Han, Mohan Bai, Yanting Chen, Yali Gong, Ming Wu, Hefa Yang, Qing Chen, Ting Xu, Yuquan Wei, Guochun Ding, Ji Li

**Affiliations:** 1 College of Resources and Environmental Science, Beijing Key Laboratory of Biodiversity and Organic Farming, China Agricultural University, Yuanmingyuan West Road No.2, Haidian District, Beijing 100193, China; hh071@163.com (H.H.); baimohantju@163.com (M.B.); chenyanting@cau.edu.cn (Y.C.); gongyali0104@126.com (Y.G.); cau188ming@cau.edu.cn (M.W.); qchen@cau.edu.cn (Q.C.); xuting@cau.edu.cn (T.X.); weiyq2019@cau.edu.cn (Y.W.); 2 College of Life Science, Langfang Normal University, Langfang 065000, China; 3 Organic Recycling Institute (Suzhou) of China Agricultural University, Wuzhong District, Suzhou 215128, China; 4 Quzhou Experimental Station of China Agricultural University, Quzhou 057250, China; yanghefayou@163.com

**Keywords:** compost, sulfadiazine resistance, high throughput sequencing, *sul1*, *sul2*

## Abstract

Although composting is effective in deactivating antibiotic substances in manure, the influence of compost fertilization on the occurrence and dissemination of antibiotic resistance in arable soils remains to be controversial. Herein, the abundance and diversity of two sulfonamide resistance genes (*sul1* and *sul2*) in soil fertilized by compost spiked with two concentrations of sulfadiazine (1 and 10 mg kg^−1^) were studied intensively by qPCR and high throughput sequencing based on a two-month microcosm experiment. The concentration of sulfadiazine decreased rapidly after spiking from 25% at Day 1 to less than 2.7% at Day 60. Relative abundance of both *sul1* and *sul2* were significantly higher in soil amended with compost than the non-amended control at Day 1 and slightly decreased with incubation time except for *sul2* in the S10 treatment. Soil bacterial communities were transiently shifted by compost fertilization regardless of the presence of sulfadiazine. Relative abundance of genera in three hubs positively interlinked with *sul1* and *sul2* were significantly higher in compost treated soil than the control at Day 1, 7 and 21, but not at Day 60. High throughput sequencing analyses revealed that most detected (>67% in relative abundance) *sul1* and *sul2* genotypes sharing >99% similarity with those found in gammaproteobacterial pathogens frequently were commonly present in compost and soil. These results indicated that compost fertilization might increase the abundance rather than diversity of sulfadiazine-resistant populations in soil, which may be facilitated by the presence of sulfadiazine.

## 1. Introduction

A large number of antibiotics are being used as precaution and therapy in industrial high-density animal farms worldwide. In China, the consumption of different antibiotics reached approximately 162,000 tons in 2013 [1]. Sulfonamide, fluoroquinolones, macrolides, β-lactams and tetracycline were the most used in livestock [1,2]. Many antibiotics such as sulfonamides cannot be absorbed by animals and are largely excreted via urine or feces. They are also not or only to a low level degraded during manure storage and may pose a selective pressure on antibiotic resistant bacteria. Ten to ten thousand folds’ elevation of antibiotic-resistance in manure has been reported [3]. Application of manure containing antibiotic-resistant bacteria, residuals antibiotics and potential pathogenic bacteria may facilitate the spread of antibiotic resistance genes (ARGs) into human or animal pathogens, which pose a huge threat to public health [4,5,6,7,8]. A recent study also found that tetracycline and sulfamerazine introduced via manure into soil might be accumulated in *Zea mays* L. [9]. The presence of antibiotics in manure also induced changes in soil microbial communities and shifted taxa known as human pathogens [10,11]. Several treatments such as elongation of storage, acidification [12], mesophilic digestion [13] and composting [14] have been evaluated for their effects on the mitigation of ARGs or mobile genetic elements in manure. Among these methods, thermophilic aerobic fermentation and thermophilic anaerobic digestion tend to be more effective in deactivating several antibiotics (such as oxytetracycline, macrolide and fluoroquinolone) and ARGs than the corresponding mesophilic treatments [14,15]. However, persistence or elevation of ARGs abundance was still observed in arable soils fertilized by manure or composts [3,16,17]. Relative abundance of two tetracycline resistance genes (*tetM* and *tetK*) largely flocculated over growing seasons in a two-year field experiment [18]. Arable soil was regarded as a receptor of manure, and its ARGs, antibiotics and antibiotic residuals served as a reservoir of drug-resistant bacteria, which could be transferred through food chains and other environmental routes [19,20]. Recently, manure-borne microorganisms were suggested to be contributed at a large extent to the elevation of ARGs in manured soil [21]. Several other studies also demonstrated that the majority of microorganisms inhabiting organic fertilizer may not fit for soil environments [22,23], suggesting that the remaining antibiotics in organic fertilizer may be the cause of elevated antibiotic resistance in arable soils. 

In the present study, a microcosm experiment with four treatments was conducted to study the effects of compost application and sulfadiazine on soil microbiome and sulfonamides resistant populations. Four treatments included soil (CK), soil amended with compost (S0) and soil amended with compost containing 1 (S1) or 10 (S10) mg kg^−1^ of sulfadiazine. Dynamic of sulfonamides-resistant populations and total soil microbial population were evaluated by real-time quantitative PCR and Illumina sequencing of *sul1*, *sul2* and 16S *rRNA* amplicons. This study might provide an in-depth understanding of the shift of sulfadiazine resistome in soil under antibiotic stress or fertilized by compost.

## 2. Materials and Methods

### 2.1. Microcosm Experiment

The silt loam soil from a long-term greenhouse experiment was used for the microcosm experiment. All soil was immediately passed through a 2 mm mesh sieve to remove plant debris and stones immediately after sampling in Dec 2014. Then it was stored in an incubator (30 °C) for 10 days for equilibration before the experiment. Four treatments were prepared as follows: 1-kilogram soil (dry weight) was amended with 140 g of compost (S0) or the same amount of compost pre-mixed with sulfadiazine, and soil without compost and sulfadiazine amendment (CK) served as a control. The seeding compost was prepared as follows: 270 g of fresh compost was sprayed with sulfadiazine solutions (50 mL with concentrations of 0, 0.5 and 5 g L^−1^, respectively) in a closed container, which was shaken vigorously to reach a final concentration of 1 (S1) and 10 (S10) mg kg^−1^. The amount of compost used was similar to that in the field for spring vegetables in 2014. Five independent replicates for each treatment at each sampling time were included. After mixing, 45 g of soil were redistributed in a 50 mL conical tube, and all tubes were randomly placed and incubated in the dark at 30 °C for 60 days. The moisture of all soil was adjusted to 40%, which was comparable with field conditions by adding sterilized deionized water during the experiment period. A total of 80 soil samples were taken on Day 1, 7, 21 and 60 (5 replicates per treatment × 4 treatments per sampling × 4 samplings).

### 2.2. Sulfadiazine Quantitation

Concentrations of sulfadiazine in soil and compost were analyzed according to the previous method [24]. Fresh soil and compost samples were lyophilized by a vacuum freeze drier, then sieved through a 2-mm mesh and stored in the dark at room temperature until analysis. Samples were extracted by adding multiple extracting agents (e.g., EDTA-Mcllvaine buffer, methanol and acetone) and ultrasound-assisted. The standard solutions of sulfadiazine were made using a 10-fold series dilution of stock solution (10 mg mL^−1^) for five gradients and stored at 4 °C. The analysis was performed using an Agilent HPLC-MS system (Waters, Milford, MA, USA). Peak areas of sulfadiazine for each sample and standard solutions were recorded for further calculations.

### 2.3. TC-DNA Extraction, Real Time qPCR of sul1, sul2 and 16S rRNA Gene

Total community DNA was extracted from 0.5 g of soil or compost using FastDNA spin Kit for soil (MP, Biomedicals, Santa Ana, Carlsbad, CA, USA). Quantification of 16S *rRNA* genes and sulfadiazine resistance genes *sul1* and *sul2* were performed according to previous studies [25,26,27]. For *sul1* and *sul2*, a 50 μL reaction volume contained 2.5U Taq DNA polymerase and 25 μL buffer; both were made by TaKaRa (Bao-TaKaRa company, Dalian, China), 0.5 μL of each primer (10 μM), 0.5 μL probe (10 μM), 2.5 μL BSA (0.5%) and 5 μL template. Thermocycles for *sul1* were 5 min at 94 °C and 40 cycles consisting of 15 s at 95 °C, 1 min at 60 °C, while for *sul2* were 5 min at 94 °C and 40 cycles consisting of 15 s at 95 °C, 15 s at 53 °C, 1 min at 60 °C. The sequence of all primers and Taqman probes of *sul1* and *sul2* were given in Table 1. Real-time qPCR reactions were performed in an iQ-5 real-time PCR detection system (Bio-Rad, Hercules, California, USA). R^2^ values were more than 0.99, and the amplification efficiencies ranged between 84% and 98%. Gene copy numbers of *sul1* and *sul2* were adjusted to 16S *rRNA* for further analysis. One-way ANOVA in conjunction with Tukey’s honest significant difference (HSD) test (*p* < 0.05) was used to compare different treatment and sampling times.

### 2.4. High Throughput Sequencing Analysis of Bacterial sul1, sul2 and 16S rRNA Gene Amplicon

Fragments of *sul1* and *sul2* were amplified with barcode-fused primers used in the qPCR analyses. Due to the length of *sul1* or *sul2* amplicon being shorter than 250 bp (the read length of illumine sequencing), an *in-silicon* PCR was performed to extract the proper fragment from each read. Then, the reads were assigned to each sample based on barcode sequences, and the primer regions were trimmed. A standalone BLASTP analysis was used to identify the translation frames, and only those sequences where the deduced ammonia acid sequence has no stop codon were included for further analysis. The *sul1* or *sul2* sequences were assigned to different genotypes based on the deduced ammonia acid sequence using usearch software [28]. The amplification, purification, sequencing and analysis of the 16S *rRNA* gene were performed according to previous descriptions [29,30,31,32,33,34,35]. All sequences have been submitted to GenBank (SRP126466). 

Beta diversity of microbial community was compared using or PCoA based on the Bray–Curtis distance. Chao1 was calculated by 100 times of re-sampling an equal number of sequences from each sample using R-add-on vegan packages to attenuate the biases caused by different read numbers [34]. The relative abundance of bacterial taxa, *sul1* and *sul2* genotype were calculated by dividing the read number for each taxon or genotype with the total read number for each sample. Co-occurrence network analysis was performed based on the Spearman correlation (cor > 0.6, *p* < 0.001). The network was analyzed with the software, Gephi [36]. Microbial hubs that were significantly different on the relative abundance were identified by a generalized linear model for binominal data using the R add-on package “multicomp” [37]. All statistical analyses and plots were performed with the software R 3.1.2 (http://www.r-project.org/, downloaded in 2020), and these tools mentioned above have been implemented into a galaxy instance (www.freebioinfo.org, processed data from January to March of 2021).

## 3. Results

### 3.1. Concentration of Sulfadiazine

**Figure 1:**
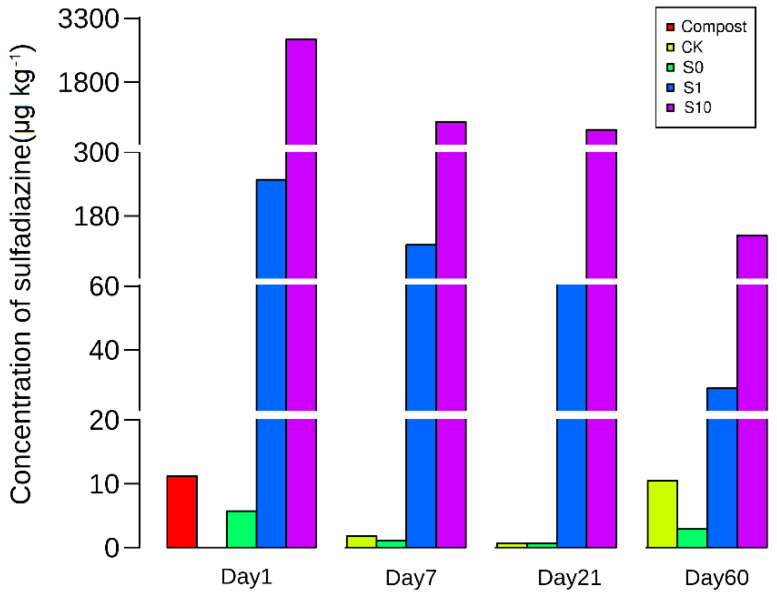
The concentration of sulfadiazine in compost and soils in different treatments. CK: soil; S0: soil amended with compost; S1: soil amended with compost and 1 mg kg^−1^ of sulfadiazine; S10: soil amended with compost and 10 mg kg^−1^ of sulfadiazine.

A small amount of sulfadiazine was also detected from soil (1.8 μg kg^−1^) and compost (11.2 μg kg^−1^) (Figure 1). The concentration of sulfadiazine decreased rapidly after spiking (Figure 1). On Day 1, the concentrations of sulfadiazine were only 251 and 2596 µg kg^−1^ in S1 and S10 soils, respectively, accounting for ca 25% of the concentration spiked (Figure 1). The concentrations rapidly decreased to 121 and 782 µg kg^−1^ at Day7 and 51 and 607 µg kg^−1^ at Day21 (Figure 1). The concentrations of sulfadiazine were only 27.1 and 140.2 µg kg^−1^ in S1 and S10 soils at Day 60, respectively, accounting for 2.7% and 1.4% of the concentration spiked (Figure 1).

### 3.2. Abundance of Bacterial sul1 and sul2 Genes

**Figure 2:**
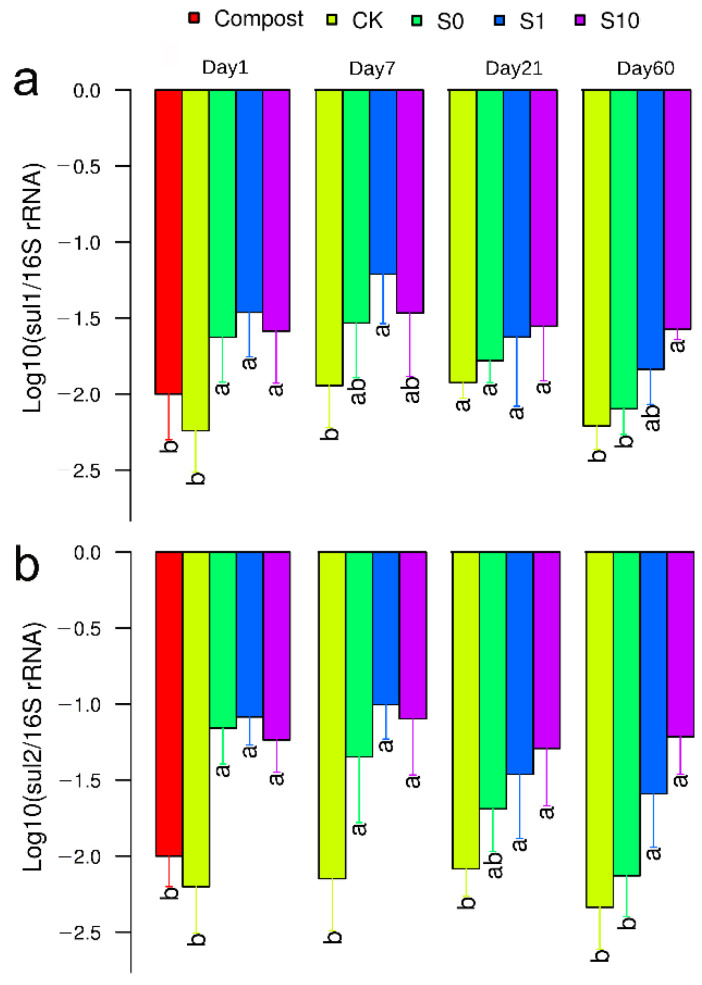
The relative abundance of *sul1* (**a**) and *sul2* (**b**) in different treatments. CK: soil; S0: soil amended with compost; S1: soil amended with compost and 1 mg kg^−1^ of sulfadiazine; S10: soil amended with compost and 10 mg kg^−1^ of sulfadiazine. The different letters above the columns in the same sampling indicate significant differences (*p* < 0.05) between treatments.

The copy numbers of 16S *rRNA* genes in different soils were comparable among all treatments over two months (Supplementary Materials, Figure S1). The relative abundance of *sul2* was significantly higher in those soils amended with compost than the non-amended control at Day 1 and 7 (Figure 2b). Interestingly, a decrease in *sul2* with incubation time was only observed for S0 and S1 but not for S10 (Figure 2b), suggesting that a higher concentration of sulfadiazine in soil may facilitate the persistence of resistant bacteria in soils. The relative abundance of the *sul1* gene tended to be lower in soils amended with compost than the non-amended control (Figure 2a). Again, a slight decrease in *sul1* with the incubation time was also observed for S0 and S1, but not for S10 (Figure 2a).

### 3.3. Bacterial Community Composition

**Figure 3:**
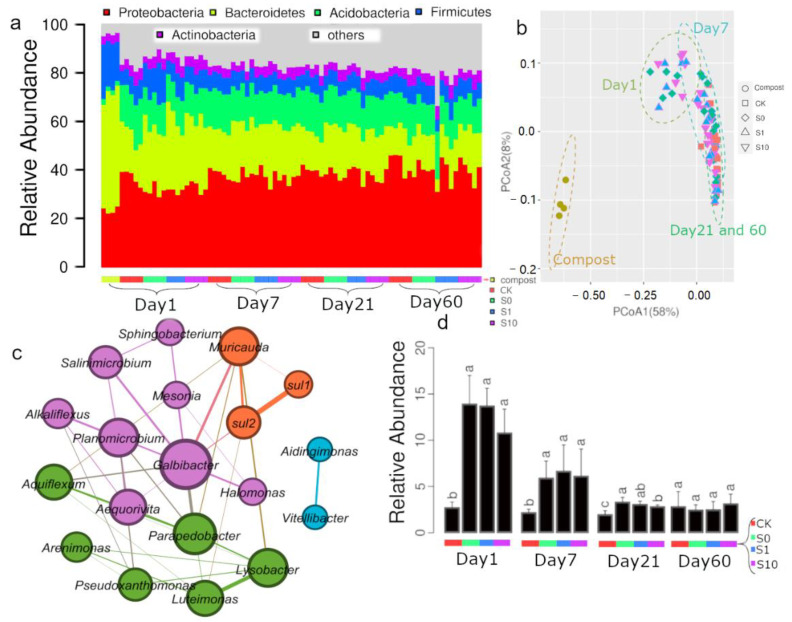
The relative abundance of dominant compost bacteria (**a**), PCoA (principal coordinate analysis) of bacterial community (**b**), the co-occurrence network between *sul1*, *sul2* and bacterial taxa (**c**) and the relative abundance of the three interlinked hubs in different treatments (**d**). CK: soil; S0: soil amended with compost; S1: soil amended with compost and 1 mg kg^−1^ of sulfadiazine; S10: soil amended with compost and 10 mg kg^−1^ of sulfadiazine.

In contrast to *sul1* or *sul2*, bacterial communities were dramatically different between compost and soil. Bacteroidetes (52.4%), Proteobacteria (21.5%), Firmicutes (17.7%), Actinobacteria (3.0%) and Acidobacteria (2.2%) were most detected phyla from compost (Figure 3a). Relative abundance of Bacteroidetes and Firmicutes was significantly higher in compost than soil, in contrast to Proteobacteria, Acidobacteria, Chloreflexi, Planctomycetes and Nitrospirae (Figure 3a). Interestingly, the relative abundance of Firmicutes in soils fertilized by compost decreased rapidly with incubation time and was comparable to the non-fertilized soil at Day 7 (Figure 3a). While the relative abundances of Bacteroidetes were comparable between compost fertilized and non-fertilized soil at Day 60 (Figure 3a). Principal coordinate analysis (PCoA) confirmed that the bacterial community was largely shaped by compost fertilization and incubation time (Figure 3b). While the similarity in community composition between compost fertilized and non-fertilized soil increased with incubation time (Figure 3b), suggesting that soil bacterial communities were resilient to the perturbation by compost fertilization.

Co-occurrence network analysis was applied to study the correlation between *sul1* or *sul2* and bacterial taxa. The majority of these positively correlated genera were affiliated with Bacteroidetes (*Aequorivita*, *Alkaliflexus*, *Aquiflexum*, *Galbibacter*, *Mesonia*, *Muricauda*, *Parapedobacter*, *Salinimicrobium*, *Sphingobacterium* and *Vitellibacter*) and Proteobacteria (*Aidingimonas*, *Arenimonas*, *Halomonas*, *Luteimonas*, *Lysobacter* and *Pseudoxanthomonas*) (Figure 3c). *Sul1*, *sul2* and these positively correlated genera formed four hubs, and three hubs were positively interlinked (Figure 3c). Both *sul1* and *sul2* were positively correlated with the genera *Muricauda* (Figure 3c). Additionally, *Galbibacter* and *Lysobacter* were also significantly correlated with *sul2* (Figure 3c). Relative abundances of three interlinked hubs were significantly higher in compost treated soil than the control at Day 1, 7 and 21 (Figure 3d).

### 3.4. Diversity of sul1 and sul2 Genes

**Figure 4:**
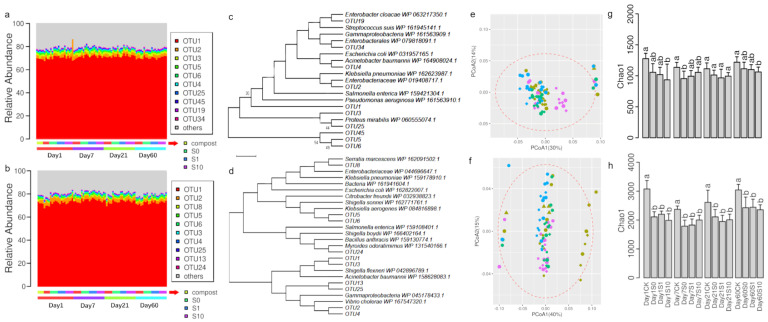
The relative abundance of most detected genotype for *sul1* (**a**) and *sul2* (**b**) gene, BLASTP analysis of *sul1* (**c**) and *sul2* (**d**) gene, PCoA (principal coordinate analysis) of *sul1* (**e**) and *sul2* (**f**) gene composition, Chao1 index of *sul1* (**g**) and *sul2* (**h**) in different treatments. CK: soil; S0: soil amended with compost; S1: soil amended with compost and 1 mg kg^−1^ of sulfadiazine; S10: soil amended with compost and 10 mg kg^−1^ of sulfadiazine.

Both *sul1* and *sul2* gene fragments were subjected to Illumina Hiseq 2500 analyses. Totally 6,475,646 and 5,236,210 sequences were acquired for *sul1* and *sul2,* respectively. The detected diversities of *sul1* and *sul2* genes were extremely high with 64,148 and 59,461 unique putative amino acid sequences, respectively. Interestingly, the most detected genotype accounted for 67.8–74.5% for *sul1* and 68.0–77.3% for *sul2* (Figure 4a,b). BLASTP analysis revealed that the deduced amino acid sequence of most detected *sul1* (OTU1) shared >99% similarity with those genes encoded within genomes of *Salmonella enterica*, *Klebsiella pneumonia* or *Escherichia coli* (Figure 4c). The most detected *sul2* genotype (OTU1) was similar (>99%) to those bacteria carried by *Shingella boydii*, *Acinetobacter Baumannii* (Figure 4d). Other most detected genotypes were also similar (>97% similarity) to *sul1* or *sul2* genes carried by those Gamma proteobacteria (Figure 4c,d). The composition of *sul1* or *sul2* genes was highly similar between compost or soils (>78% similarity) (data not shown). Alpha-diversities of *sul1* tended to be higher in control soil than those compost fertilized soils (Figure 4e). However, the significant differences were only detected between CK and S10 at Day 1 and 60 or between CK and S0 at Day 7 (Figure 4e). The alpha-diversity of *sul2* was significantly higher in CK than compost fertilized soil at all samplings (Figure 4f), suggesting slight effects of composting fertilization on alpha-diversity of *sul1* and *sul2*. No effect of sulfadiazine spiking on the alpha-diversity of *sul1* and *sul2* was detected (Figure 4e,f).

## 4. Discussion

Antibiotic resistance genes were widespread in environmental bacteria. Several antibiotic resistance genes were ubiquitous in environments, and some of which were believed to be pristine from antibiotic contamination [38]. For example, a *Paenibacillus* bacterium isolated from an underground cave that is believed to be isolated from the surface for over 4 Myr is resistant to most clinically used antibiotics [39]. A large-scale survey also revealed that the relative abundance of sulfonamide resistance genes ranged from 10^−6^ to 10^−2^ gene copies per 16S *rRNA* gene copies in the arable soils of China [40]. The functional metagenomic analysis also revealed that diverse sulfonamides resistance genes were also detected from different soil microbial communities, indicating that sulfonamides resistance was ubiquitously present in several soil environments [41]. In vitro studies have long demonstrated that the spreading of antibiotic resistance among bacteria could be strengthened under the selection pressure of antibiotics [42,43]. However, bacteria carrying antibiotic resistance genes may need more energy to replicate their ARGs during reproduction, which might be a disadvantage if there were no selective pressure from antibiotics [44]. Thus, the fate of antibiotic resistance genes in environmental samples remains to be elusive. 

### 4.1. Diversities of sul1 or sul2 Were Extremely High but Only Few Common Dominant Genotypes were Prevalent in Soil or Compost

Herein, we employed Illumina sequencing to analyze PCR amplicons of *sul1* and *sul2* genes, and the acquired diversities of both *sul1* and *sul2* genes were extremely high in soil and compost samples, suggesting that both soil and compost are reservoir rich in diverse sulfonamide resistance genes. These findings indicated that resistance to antibiotics might evolve rapidly in Bacteria [45], which may exchange with one another antibiotic resistance gene by horizontal gene transferring mechanisms [46,47] or mutate its own genes to become resistant [48]. Several environmental stressors such as starvation, antimicrobials may drive the evolution of antibiotic resistance [49,50,51] or contribute to their maintenance in environments [14,47]. Although sequencing errors may cause artificial diversity [52], we analyzed the dataset in a stringent manner by checking primer region, translation frame and deduced amino acids. In contrast, it is still possible that novel *sul1* or *sul2* genes may not be undetected due to that the spectrum of genes that can be amplified has been defined by the primer sequences. Recently, researchers detected novel sulfonamide resistance genes which shared relatively low similarity with known entities in reference database via metagenomics analysis, highlighting a requirement of extensive study on environmental resistomes [41]. 

Despite the immense diversities of *sul1* or *sul2*, the community compositions were highly similar between compost and soil, of which distinct bacterial communities were detected. It is likely due to that the most detected genotypes of *sul1* or *sul2* (accounting for more than 67%) were commonly present in both compost and soil, and their proportions were not affected by compost fertilization, sulfadiazine spiking or incubation. These genotypes were highly similar to those hosted by species such as *Salmonella enterica*, *Klebsiella pneumonia*, *Escherichia coli Shingella boydii* and *Acinetobacter baumannii*, which were known as pathogenic bacteria [53,54,55,56]. It is also worth noting that all these species except for *Klebsiella pneumonia* (relative abundance <0.03%) were not detected from both soil and compost by the 16S *rRNA* gene analysis. Since both *sul1* and *sul2* genes were reported to be present on plasmids [57,58], which could spread into indigenous soil microorganisms [46,47]. In consideration of the relative abundance of *sul1* and *sul2* by qPCR, these data suggested the dominant *sul1* or *sul2* genotypes were possibly present in a wide spectrum of different taxonomic groups. However, the spectrum of their hosts and which mechanisms drive the dominance of these genotypes in different environmental bacteria remains to be explored.

### 4.2. Compost Fertilization Elevated the Abundance of sul1 and sul2 in Soils, and the Co-Introduced Sulfadiazine may Facilitate the Persistence of Such Resistance

Quantitative PCR analysis revealed that *sul1* and *sul2* genes were significantly higher in the compost treated soils than the control shortly after the fertilization. This result indicated that compost amendment possibly stimulated the growth of bacteria carrying *sul1* or *sul2* genes. In general, these findings agree with other studies that ARGs in soil were frequently elevated after amendment with an organic fertilizer such as manure or compost [3,18,21,46]. That transient enrichments of sulfonamide or other antibiotic resistance with different manure applications without known selective pressure were also observed in other studies [21,59]. However, repeated application of manure containing antibiotics could cause an increase in antibiotic resistance in soil [46]. Herein, the decrease in *sul1* or *sul2* with incubation was slower in S10 than in other treatments. Thus, the presence of sulfadiazine may facilitate the persistence of *sul1* or *sul2* in soils. In the other aspect, sulfadiazine might be not a long-living persistent selective pressure for *sul1* or *sul2* as its concentration decreased dramatically with incubation time (Figure 1). Despite sulfonamide antibiotics were adsorbed by soil or compost [60,61], the sorption coefficients were very low, indicating that these substances were highly mobile [62].

### 4.3. Resilience of Soil Bacterial Community to the Perturbation of Compost 

Similar to other organic fertilizers, compost application not only introduced a complex of nutrients or carbons but also exogenous microbial communities into the soil. The bacterial compositions in compost dramatically differed from those in soil. Those soil bacterial communities shifted by compost rapidly become to be similar to the control, indicating that bacteria in compost may diminish after entering into the soil (Figure 4a). A previous study demonstrated that indigenous soil microorganisms inhibited the invasion and establishment of exogenous soil microorganisms from manure [21]. Co-occurrence network analysis also suggested that both *sul1* or *sul2* genes were positively correlated with several genera, which were also enriched after compost fertilization (Figure 3c). Taken together, these results suggested that compost fertilization may trigger the growth of indigenous soil microorganisms carrying sulfonamide resistance.

## 5. Conclusions

Compost fertilization triggered a transient increase in sulfonamides resistant bacteria in soil, and the presence of sulfadiazine might facilitate the persistence of resistance populations. These findings highlight the importance of deactivating antibiotics or other selective pressure on mitigating ARGs spreading in agricultural systems. The dominant genotype of *sul1* and *sul2* might be widely distributed in different bacteria inhabiting in soil and compost and have evolved into huge genetic diversity.

## Figures and Tables

**Table 1:** Probes and primers used for the real-time qPCR.

Genes	Sequences of Probes	Sequences of Forward Primer (5′-3′)	Sequences of Reverse Primer (5′-3′)	References
16S *rRNA*	CTTGTACACACCGCCCGTC	CGGTGAATACGTTCYCGG	GGWTACCTTGTTACGACTT	[25]
*sul1*	CAGCGAGCCTTGCGGCGG	CCGTTGGCCTTCCTGTAAAG	TTGCCGATCGCGTGAAGT	[26]
*sul2*	CGGTGCTTCTGTCTGTTTCGCGC	CGGCTGCGCTTCGATT	CGCGCGCAGAAAGGATT	[27]

## Data Availability

The data presented in this study are available in GenBank (SRP126466) or freebioinfo.org.

## Supplementary Materials

The following are available online at https://www.mdpi.com/2079-6382/10/6/699/s1, Figure S1: The abundance of bacteria in different treatments.
