# Supplementary material for: Dynamics of Diversity and Abundance of Sulfonamide Resistant Bacteria in a Silt Loam Soil Fertilized by Compost
Source: Antibiotics (Basel). 2021 Jun 11;10(6):699. doi: 10.3390/antibiotics10060699 (PMC8230599; doi:10.3390/antibiotics10060699)
Supplement: Supplementary file 1 [file antibiotics-10-00699-s001.zip › antibiotics-1174559-supplementary.pdf]

antibiotics

MDPI

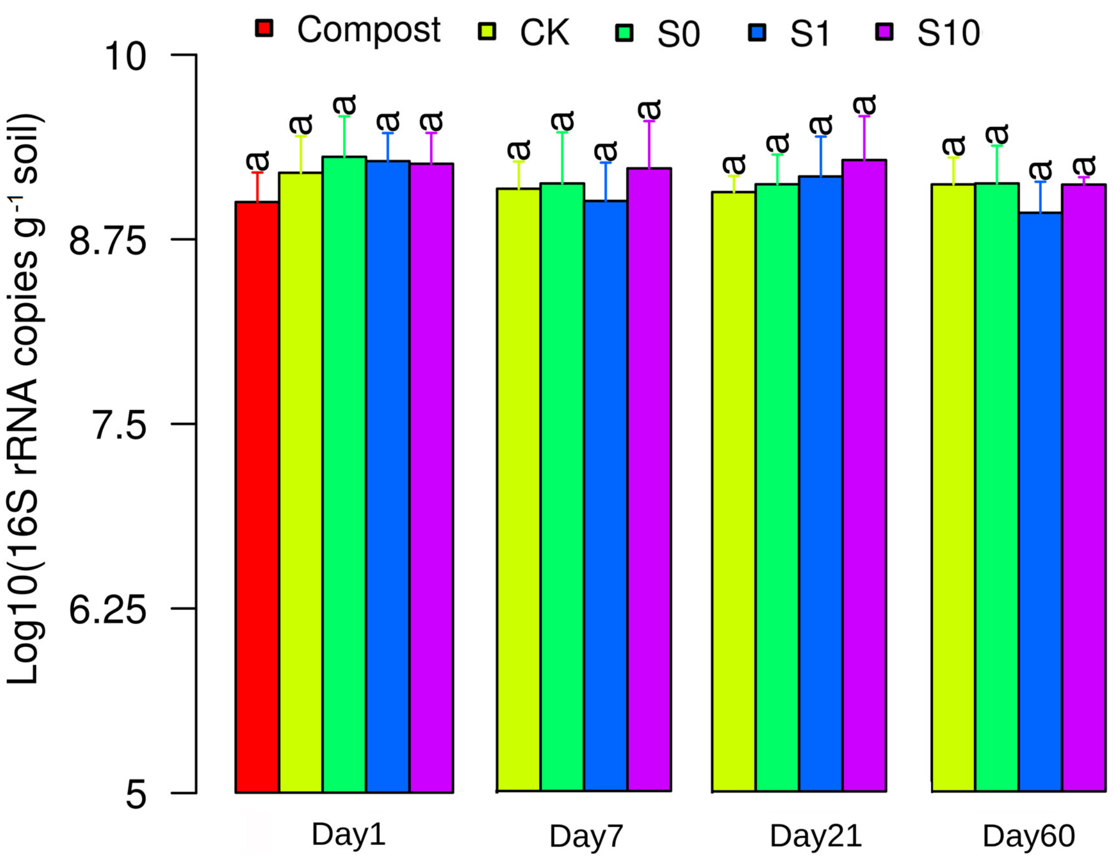

Figure S1. The abundance of bacteria in different treatments. CK: soil; S0: soil amended with compost; S1: soil amended with compost and 1 mg kg-1 of sulfadiazine; S10: soil amended with compost and 10 mg kg-1 of sulfadiazine. The same letters above the columns in the same sampling indicate no significant differences ($p > 0.05$) between treatments.
